# Diagnostic Methods for Feline Coronavirus: A Review

**DOI:** 10.4061/2010/809480

**Published:** 2010-07-28

**Authors:** Saeed Sharif, Siti Suri Arshad, Mohd Hair-Bejo, Abdul Rahman Omar, Nazariah Allaudin Zeenathul, Amer Alazawy

**Affiliations:** Department of Veterinary Pathology and Microbiology, Faculty of Veterinary Medicine, Universiti Putra Malaysia, Serdang, Selangor D. E. 43400, Malaysia

## Abstract

Feline coronaviruses (FCoVs) are found throughout the world. Infection with FCoV can result in a diverse range of signs from clinically inapparent infections to a highly fatal disease called feline infectious peritonitis (FIP). FIP is one of the most serious viral diseases of cats. While there is neither an effective vaccine, nor a curative treatment for FIP, a diagnostic protocol for FCoV would greatly assist in the management and control of the virus. Clinical findings in FIP are non-specific and not helpful in making a differential diagnosis. Haematological and biochemical abnormalities in FIP cases are also non-specific. The currently available serological tests have low specificity and sensitivity for detection of active infection and cross-react with FCoV strains of low pathogenicity, the feline enteric coronaviruses (FECV). Reverse transcriptase polymerase chain reaction (RT-PCR) has been used to detect FCoV and is rapid and sensitive, but results must be interpreted in the context of clinical findings. At present, a definitive diagnosis of FIP can be established only by histopathological examination of biopsies. This paper describes and compares diagnostic methods for FCoVs and includes a brief account of the virus biology, epidemiology, and pathogenesis.

## 1. Introduction

Feline coronaviruses (FCoVs) are enveloped viruses with a large, capped, polyadenylated RNA genome of about 29,190 nucleotides. The FCoVs are group 1 coronaviruses, recently designated as members of subgroup 1a in the family *Coronaviridae*. Other members of this subgroup include transmissible gastroenteritis virus (TGEV), canine coronavirus (CCV), raccoon dog coronavirus (RDCoV), and Chinese ferret badger coronavirus (CFBCoV) [1].

The order of the genes encoding the viral polymerase (Pol) and the four structural proteins (the spike, envelope, membrane, and nucleocapsid proteins) is 5′-Pol-S-E-M-N-3′. These genes are present in all coronaviruses. The feline coronavirus genome also includes additional genes (3a, 3b, 3c, 7a, and 7b) that encode nonstructural proteins. The functions of these gene products are not fully understood [2].

Two biological types of FCoVs are known: feline infectious peritonitis virus (FIPV) and feline enteric coronavirus (FECV). In the widely accepted “*in vivo* mutation” theory, FIPV arises by mutation from parental FECV in the gastrointestinal tract of an infected cat, spreads systemically and causes FIP [3–5]. The mutation sites are not fully understood, but some accessory genes (3c and 7b) are candidates for the site of the critical mutations responsible for FIP [6, 7]. An alternative hypothesis is the “circulating virulent/avirulent” theory, which suggests that both virulent and avirulent strains circulate in cat populations, and susceptible individuals exposed to the virulent strains develop the disease. This hypothesis was proposed after sequence analysis of four genes (Pol, S, M and 7b) from FCoV-infected healthy cats and cats with FIP. Phylogenetic analyses revealed that sequences of the M and 7b genes in viruses obtained from healthy cats were distinct from those obtained from sick cats and suggest the coexistence of both biotypes in cats [8]. However, as these viruses undergo mutation readily [7] and genetic differences in the 7b gene were not correlated with pathogenicity in another study [9], the epidemiology of FIPV is yet to be clarified.

Regardless of the source of FIPV and uncertainty about the significance of genetic differences, the relationship between virulence and macrophage/monocyte tropism has been firmly established [7]. While both FIPV and FECV may cause viraemia [10–12], only FIPV replicates in macrophages and causes the disease [5, 13]. Complex immune reactions between the virus, antiviral antibodies, and complement cause disseminated vasculitis, which is the hallmark of FIP [14, 15].

Based on their antigenic relationship with canine coronavirus, sequence analyses of the S gene, and their growth characteristics *in vitro*, FCoV strains can be classified into serotypes I and II. FCoV serotype I strains are wholly feline. They are difficult to grow in cell culture and cause a slowly developing cytopathic effect. FCoV serotype II strains seem to have arisen by recombination between FCoV serotype I and CCV. They grow more rapidly than serotype I viruses and induce a lytic cytopathic effect. FIPV and FECV strains can be serotype I or II [6, 16–18].

FCoV infection is extremely common in cat populations. Antibodies against FCoV are found in 20%–60% of pet cats and up to 100% of cats in catteries or multi-cat households [14, 19–24]. FCoVs are highly infectious and spread predominantly by the faecal-oral route. About 75%–100% of cats in multi-cat environments shed the virus [14, 25, 26].

## 2. Diagnosis of FCoV

FECV infections are usually associated with mild disease at most. Many cases remain asymptomatic, and in young kittens mild transient diarrhoea of several days duration is generally the only sign. Vomiting occurs in a smaller proportion of cases and is not usually a prominent feature. Infection with FECV rarely causes disease of sufficient severity to require specific diagnosis of the underlying aetiological agent. The virus can be demonstrated in the faeces of infected kittens by electron-microscopic examination or by reverse transcriptase polymerase chain reaction (RT-PCR) assay. However many healthy cats and kittens will also shed FCoV in their faeces. Thus, other than for detection of carriers or demonstrating the presence of FCoV infection in a colony of cats, such investigations have limited value [15, 27].

FIPV variants of FCoV cause fatal peritonitis. Cats with a poor cell-mediated immune response develop the effusive or “wet” form of disease, which is an immune complex vasculitis that causes leakage of protein-rich fluid from the blood vessels into the abdominal cavity, leading to a distended abdomen. In cats with partial cell-mediated immunity, the non-effusive or “dry” form develops, with pyogranulomatous or granulomatous lesions in multiple tissues. Dry FIP may become effusive in the terminal stages of the disease, when the immune system collapses [28, 29]. 

Ante-mortem diagnosis of FIP is difficult and frustrating. Difficulties in definitively diagnosing FIP arise from the lack of specific clinical signs, the lack of pathognomonic biochemical abnormalities, and the low sensitivity and specificity of tests routinely used in practice. FIP diagnosis is based on assimilation of the history, haematology, and other supportive diagnostic tests, including serology and findings from imaging, tissue biopsies, and PCR [8, 15]. A typical history of FIP cases includes acquisition of the cat from a cattery and a fever that waxes and wanes and does not improve with antimicrobial therapy. Most commonly, kittens are infected between the ages of 6 and 8 weeks, at a time when maternally derived antibodies wane, mostly through contact with faeces from their mothers or other FCoV-excreting cats [27].

**Figure 1:**
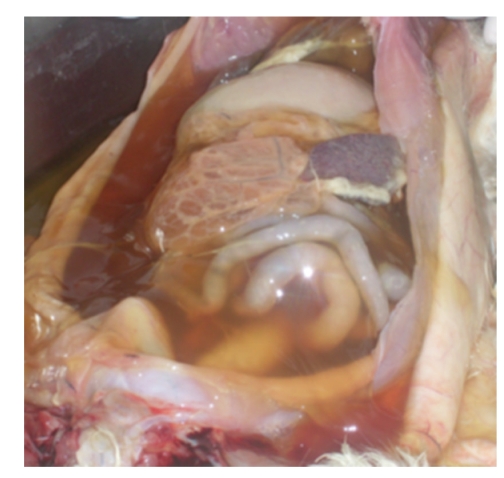
Peritoneal cavity of a cat with effusive FIP. A considerable amount of ascitic fluid can be seen in the abdominal cavity.

**Figure 2:**
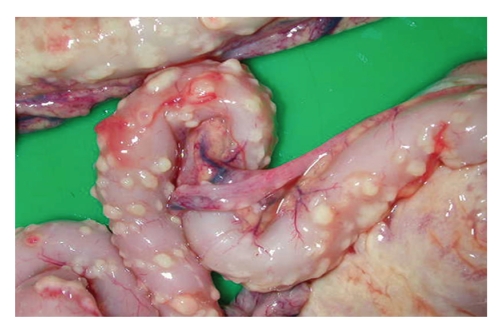
Intestines of a cat with effusive FIP. Pyogranulomatous foci are seen as punctate fibrinous plaques on the serosal surface of the intestines.

**Figure 3:**
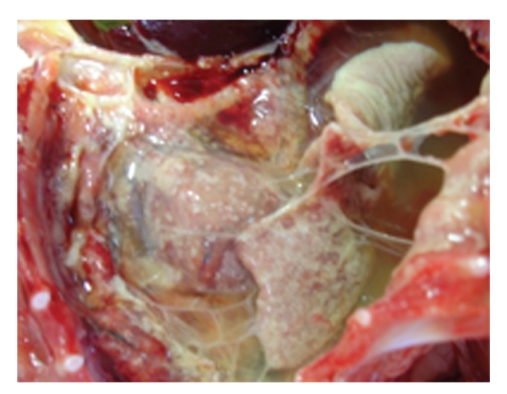
Pleural cavity of a cat with effusive FIP. Fibrinous serositis and adhesions of the lung with fibrin strands can be seen.

**Figure 4:**
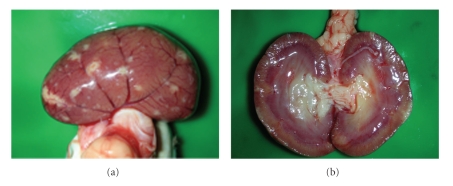
Kidney of a cat with non-effusive FIP. Granulomatous lesions can be seen on the capsular surface and in the parenchyma of the kidney.

FIP is a disease with extremely diverse clinical manifestations. In the “wet” form, the most characteristic sign is a considerable amount of intracavitary effusion (Figure 1). The typical lesions of effusive FIP are pyogranuloma and fibrinous plaques on the serosal surfaces of abdominal organs (Figures 2 and 3). Dyspnea, mild pyrexia, and muffled heart sounds are common. Ocular involvement in FIP can include uveitis, keratic precipitations, and changes in the coloration of the iris. In the non-effusive form, lesions commonly occur in eyes and CNS, but granulomas may also be found in the peritoneal cavity, leading to more diverse, and often more vague, clinical signs [15, 29] (Figure 4).

Radiographs can reveal pleural, pericardial, or peritoneal effusion and hepatomegaly or renomegaly. Mesenteric lymphadenopathy may result in abdominal mass lesions in some cats. Ultrasonography can be used to confirm the presence of abdominal fluid in cats with minimal fluid volumes and to evaluate the pancreas, liver, lymph nodes and kidneys. Magnetic resonance imaging (MRI) can reveal periventricular contrast enhancement, ventricular dilation, and hydrocephalus in cats with neurological FIP [30, 31].

## 3. General Diagnostic Tests

There are a number of laboratory findings that are common in cats with FIP, but none of them is pathognomonic. Although it is often stated that lymphopaenia and neutrophilia are typical of FIP, this change can be interpreted as a typical “stress leukogram” that occurs in many severe systemic diseases in cats [32, 33]. 

The most consistent laboratory finding in FIP is an increase in total serum protein concentration. This is found in approximately 50% of cats with effusive disease and 70% of cats without effusive disease. The increase in total protein is caused by an increased concentration of globulins, mainly *γ*-globulins. A *γ*-globulin concentration of more than 32% is characteristic of FIP [33–36]. Changes in the serum protein profile lead to a decreased albumin-to-globulin (A : G) ratio. An A : G ratio of less than 0.5 is strongly correlated with FIP [37].

Acute phase proteins (APPs) are a large and varied group of glycoproteins in the serum, concentrations of which increase (or decrease) during certain inflammatory disorders. The concentrations of APPs such as feline *α*1-acid glycoprotein (fAGP) and serum amyloid A (SAA) can be measured and facilitate the diagnosis. SAA and fAGP concentrations increase in FIP, but they are not specific for this disease. While moderately elevated levels of acute-phase proteins are found in several inflammatory conditions, high levels of fAGP (>1.5 g/L) and SAA in plasma or effusions can indicate FIP and may be useful supportive tests [38–41]. 

Sampling from effusions is an important diagnostic step for FIP because tests on effusions have much higher diagnostic value than tests performed on blood samples. Effusions are typically clear yellow and viscous and may form fibrin strands. However, the presence of this type of fluid in body cavities alone is not diagnostic. Involvement of peritoneal and plural cavities has been reported in 58% and 11% of FIP cases, respectively [7, 15].

The effusion seen in FIP is classified as a modified transudate to exudate with a very high-protein content (>3.5 g/dL) and moderate cellular content. FIP effusions can be examined using a simple and cheap method called the Rivalta test. Adding a small drop of effusion to a test tube containing distilled water and a drop of 8% acetic acid can cause precipitation because of the high-protein content. This test seems to be useful for differentiation between effusions caused by FIP and effusions caused by other diseases. However, false-negative results can be obtained in cats with bacterial peritonitis and false-positive results in cats with lymphoma [37, 38].

Cytological evaluation of the effusion in cats with FIP reveals macrophages and neutrophils in a dense proteinaceous background. Neutrophils are non-degenerate or may show mild nuclear degenerative changes. Lymphocytes and plasma cells may also be found in the fluid.

In a study by Hartmann and colleagues [37], immunofluorescent staining of intracellular FCoV antigen in macrophages in effusions had a positive predictive value of 100%, but the negative predictive value was not high (57%). This could be due to the small numbers of macrophages in the smear or masking of the antigen by competitive binding of anti-FCoV antibodies in the effusion [15].

Other laboratory parameters (e.g., liver enzymes, bilirubin, urea, and creatinine) can be variably increased, depending on the degree and localization of organ damage, but they are not helpful in making an aetiological diagnosis [15, 27, 38].

## 4. Serology

Measurements of antibody in serum are useful diagnostic tools for detection of FCoV infection. However, since a large percentage of healthy cats have antibodies against FCoV, antibody testing is more helpful in the management of FCoV infection (e.g., creating an FCoV-free cattery) [15]. The sensitivity and specificity of a commercially available in-practice test kit for detection of FCoV antibodies (ImmunoComb FCoV Antibody Test Kit, Biogal, Israel) were 95% and 83%, respectively [42].

Antibody testing in cats suspected to have FIP has limited value in confirmation of the diagnosis and results should be interpreted carefully. Some cats with the wet form of FIP have low titres or even no antibodies against FCoV. This is because the large amounts of virus in the cat's body bind to antibodies and render them unavailable to antigen in the test or because the antibodies are lost in effusions [15]. The use of anti-7b proteins for serology does not appear to offer any significant advantage [43].

Since FIP is an immune-mediated disease, antibody-antigen complexes may circulate in the serum and effusions. The circulating complexes can be detected using a competitive enzyme-linked immunosorbent assay (ELISA). However, the utility of this assay is limited because the positive predictive value of the test is not high (67%) and there are many false-positive results [37, 44].

## 5. Reverse Transcriptase PolymeraseChain Reaction (RT-PCR) Assays

There are several reports of detection of FCoV by RT-PCR. Some used primers targeted at conserved regions of the viral genome, such as the Pol [45–47], the 7b gene [43, 48, 49], and the 3′ untranslated region (3′ UTR) [50–52]. RT-PCR assays using these primers are able to detect most, if not all, FCoV strains and could be a valuable tool for screening for the virus in cat populations. The sensitivity and specificity of the RT-PCR assay could be increased using real-time RT-PCR techniques [53]. As the sequence of the S gene differs between serotypes I and II, some RT-PCR assays have targeted the S gene to differentiate the FCoV serotypes [18, 54].

Since specific genetic determinants of FCoV biotypes are unknown and the genome contains various single nucleotide polymorphisms (SNPs) [7, 55, 56], it is not possible to design PCR primers to distinguish between FIPV and FECV [11] and thus discriminate between FIP cases and FCoV-positive healthy cats. In 2005, Simons and colleagues [57] introduced a new PCR-based approach for detection of FIP. The approach was based on the key pathogenic event in FIP, viral replication in macrophages and monocytes. The primers targeted the conserved region of the M gene and the leader sequence to detect replicating virus in the blood. The assay had high sensitivity and specificity [57] and is currently used in some diagnostic laboratories [28, 58]. However, in a study on 26 cats, Can-Şahna et al. (2007) found the specificity of the assay using same primers to be poor [59]. The reason for the high-false-positive rate in healthy cats (53%) in this second study is not clear, but the different RNA extraction kits used in these studies may have affected the quality of the template RNA and the RT-PCR outcome [47]. Moreover, since both studies used conventional RT-PCR techniques, they were not able to quantify the replicating mRNA in blood of infected cats. Therefore, a quantitative real-time RT-PCR assay that could determine the amount of viral mRNA in blood may be able to better differentiate FCoV-positive healthy cats from FIP cases.

RT-PCR assays have been used to detect FCoV in faecal samples and are sensitive and useful for documenting that a cat is shedding FCoV in faeces. Faecal samples must be carefully handled, kept frozen, and protected from the RNA-degrading enzymes that are present in most environments. RT-PCR should be performed as soon as possible after sampling, and even freezing samples may result in false negative results. The strength of the RT-PCR signal in faeces correlates with the amount of virus present in the intestine [15]. Comparisons between RNA samples extracted from faecal suspensions and FCoV-infected cell culture supernatants showed that the presence of faecal factors significantly inhibited the reverse transcription reaction [47]. However, Pedersen et al. [26], found no evidence for faecal inhibitors in their RT-PCR assay.

The virus can be detected in various tissues and ascitic fluid [50, 56, 60, 61]. Liver (48%) and spleen (42.3%) samples appear more likely to contain detectable FCoV than the kidneys (21.1%). In addition, the amounts of RNA extracted from fresh tissues were significantly higher than from tissue fixed in formalin, ethanol or Bouin's solution [61].

Primers designed to detect FIP in ill cats were also found to be able to amplify FCoV in healthy cats [12, 37, 50]. Thus, RT-PCR results should be interpreted in conjunction with the clinical status of the cat and cannot be used as the sole test to diagnose FIP. There are several plausible explanations for false-negative RT-PCR results, including degradation of RNA, failure of the reverse transcription reaction, and variation in the nucleotide sequences of FCoVs. Infection with CCV or TGEV could also contribute to false-positive results because they share similar conserved regions [8, 54].

## 6. Histopathology and Immunohistochemistry

**Figure 5:**
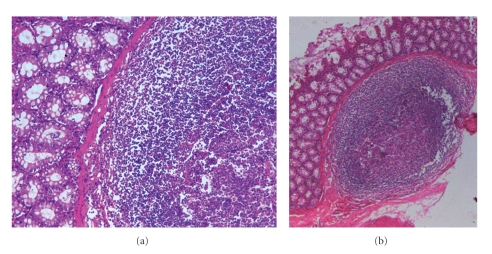
Granulomatous colitis of a cat with effusive FIP showing infiltration of inflammatory cells.

**Figure 6:**
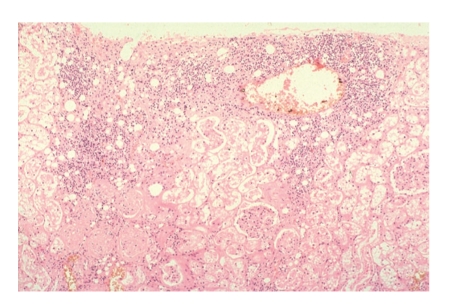
Kidney of a cat with FIP. Severe degenerative and advanced necrotic changes within the lining endothelium of the convoluted tubules (mostly cytoplasmolysis). Frank patchy interstitial nephritis, as indicated by the heavy infiltration of lymphocytes, plasma cells and some dead neutrophils, together with dilatation and congestion of the interstitial blood vessels. The photograph was kindly provided by Dr. Diane Addie.

Histopathological confirmation of FIP has been used to define cases and has been regarded as the “gold standard” for diagnostic test comparisons. Haematoxylin and eosin (H & E) stained sections typically have localized inflammation with macrophages, neutrophils, lymphocytes, and plasma cells (Figure 5). Vascular lesions may be found surrounded by proliferation of inflammatory cells and this is characteristic for wet FIP. Pyogranulomas are mainly associated with fibrinous necrosis and may be large and consolidated or numerous and small. Focal accumulations of inflammatory cells and necrotic-proliferative lesions are typical of the granulomatous lesions of dry FIP [18, 27, 58, 62] (Figure 6).

Immunohistochemical tests, such as immunoperoxidase staining, may enable the detection of FCoV antigen in tissue. Immunostaining cannot differentiate between FECV and FIPV, but as FIPV replicates more actively, higher concentrations of the viral antigen are found in FIP cases [15]. Viral antigen concentrations are lower in lesions in cats with dry FIP than in those of cats with wet FIP [14].

## 7. Conclusion

There are no pathognomonic clinical signs or specific laboratory tests for FIP in cats. The presence of antibodies does not indicate FIP and the absence of antibodies does not exclude it. Many authors agree that serological data alone have limited diagnostic value. PCR assays are able to directly detect the FCoV genome but, although they appear to be more sensitive for detection of coronaviral infection in cats, the results must be interpreted in conjunction with other clinical findings and cannot be used as the sole criterion for diagnosis of FIP. A definitive diagnosis of FIP should be confirmed by histopathology or detection of intracellular FCoV antigen by immunofluorescent or immunohistochemical staining.
